# Ability Orientation or Good Character? Moderated Mediation Mechanism to Determine the Impact of Telepresence on Consumer Purchasing Intention in Cross-Border E-Commerce

**DOI:** 10.3389/fpsyg.2022.883101

**Published:** 2022-05-06

**Authors:** Haijin Gui, Untung Rahardja, Xianchuan Yang, Yan Yan

**Affiliations:** ^1^School of Business, Wuxi Vocational Institute of Commerce, Wuxi, China; ^2^Synergetic Innovation Research Base of Digital Business Development of Jiangsu Province, Wuxi, China; ^3^Science and Technology Faculty, University of Raharja, Tangerang, Indonesia

**Keywords:** telepresence, cross-border e-commerce, customer trust, information haze, moderated mediation model

## Abstract

A research model is proposed based on the telepresence theory to determine the long-term mechanism for generating consumer purchasing intention and explore potential information haze caused by information overload. A total of 406 usable samples were collected from the consumers of several cross-border e-commerce (CBEC) platforms in China. The results showed that telepresence has a positive effect on benevolence trust, integrity trust, and ability trust. As expected, benevolence trust and ability trust were found to exhibit significant mediation effects, while integrity trust did not have a significant mediation role. The moderated mediation mechanism shows that product information (description, display, and content) restrain mediation paths. The findings provide new perspectives on the information haze caused by information overload. The results suggest that promoting an ability-oriented (ability trust) business philosophy and instilling good corporate character (benevolence trust) are vital in achieving sustainable development in CBEC platforms. Eliminating information haze could also help strengthen the activation effect of telepresence and promote the guiding role of customer trust on purchasing intention. This study's theoretical and management contributions extend our knowledge of optimizing management strategies for CBEC platforms.

## Introduction

Cross-border e-commerce (CBEC) is a new business form and model that refers to business activities (e.g., display, contact, and transactions in daily trade) in e-commerce platforms of transacting parties from different countries, relying on cross-border logistics to complete physical distribution and other related transaction processes (Baek et al., 2020; Qi et al., 2020; Zhu et al., 2020). With the sluggish growth of domestic e-commerce in recent years, CBEC is expected to become the new engine supporting the development of international trade and consumption. Global business-to-consumer (B2C) CBEC deal volume has exceeded a trillion dollars, growing by an average of 20% annually (Zhu et al., 2020; Chen and Yang, 2021). The share of CBEC in global e-commerce is expected to exceed 20% by 2022 (Zhu et al., 2019). Driven by the growth in China, the Asia-Pacific region is poised to become the world's largest CBEC market (Zhu et al., 2019).

As a new economic model, CBEC research is still in its infancy (Qi et al., 2020). Various Research Topics have previously been explored, including the factors affecting the development of CBEC platforms (Strzelecki, 2019), the formation of consumer purchasing intention (Zhu et al., 2019, 2020; Chen and Yang, 2021), the endogenous impetus of enterprise involvement in CBEC (Qi et al., 2020), and the impact of CBEC on economic growth (Falk and Hagsten, 2015; Xiao et al., 2019). While some have analyzed CBEC from the consumer psychology perspective (e.g., Mou et al., 2020b), these studies have been limited. In particular, few have analyzed risk perceptions resulting from virtual consumption scenarios that hinder consumer purchasing intention (**PI**) and behavior (Kwak et al., 2019; Baek et al., 2020). Understanding how to overcome the challenges of cyber virtuality is crucial to further developing CBEC. Therefore, presence has become a hot topic in e-commerce (Faiola et al., 2013), especially in the field of CBEC (Hassanein and Head, 2007). While previous studies have preliminarily investigated the impact of telepresence (**TELE**) on customer cognition and behavior (Ijsselsteijn et al., 2000; Mollen and Wilson, 2010), the long-term mechanism for the generation of purchasing intention affected by TELE has not been thoroughly explored (Mollen and Wilson, 2010).

Some have suggested that the long-term success of CBEC should be reflected by consumer repurchasing behavior (Hsu et al., 2015; Mou et al., 2020a). This article argues that establishing the endogenous dynamic mechanisms from the customer level is more important in driving the long-term survival and sustainable development of CBEC platforms and e-tailers. Trust is a significant construct for business relationships (Hsu et al., 2014; Bilgihan, 2016). It reflects the sense of identity, credibility, and reliability toward business partners through rational thinking and analysis (Hwang and Kim, 2007) and is the outcome of accumulated customer satisfaction (Lin et al., 2015; Escobar-Rodríguez and Bonsón-Fernández, 2017). Consequently, perceived trust can predict sustainable development in CBEC (Zheng et al., 2017) and reduce uncertainty and risks (Bilgihan, 2016), and can be used as a core indicator for the long-term success of an enterprise operation (Escobar-Rodríguez and Bonsón-Fernández, 2017). In most current literature, customer trust (TRU) is processed as a unidimensional construct (e.g., Bilgihan, 2016; Zheng et al., 2017), simplifying the formation mechanism of the trust itself and its intricate influence mechanism on purchasing intention. In this article, benevolence, integrity, and ability are selected to measure perceived trust in the provider, thereby addressing the shortcomings of the current literature. An important contribution of this study is investigating the realizing route on the long-term success of CBEC platforms. More importantly, this article aims to determine whether ability is more important than good character (benevolence and integrity).

Product information is well recognized as a significant driver in forming consumer cognition and behavior in traditional e-commerce. In previous literature, its predictive role has been stressed on consumer purchasing intention (Park et al., 2005; Han and Kim, 2019; Zhu et al., 2019, 2020; Mou et al., 2020b), while little effort has been devoted to examining the moderated effect of product information on consumer purchasing intention by drivers like TELE and perceived trust. In addition, adverse choices and moral risks are easily generated by information asymmetry (Han and Kim, 2019). Excessive information presented on CBEC platforms, especially the combined information pushed by cross-border sellers, may also create an information haze that increases risk perceptions of online shopping due to uncertainties (Mou et al., 2020a), where even experienced online consumers may feel perplexed, preventing them from making rational decisions (Pavlou et al., 2007). In this study, information haze is defined as the decision dilemma in online shopping caused by overload information, which prevents the easy discrimination between low and high quality (Mou et al., 2020a). This study was one of the first to investigate the potential moderating effect on the formation of consumer purchasing intention in CBEC by further segmenting the connotation of product information.

Given the current knowledge gaps, the main objectives of this study are as follows: (1) This study investigates the causal links among variables and examines whether the TELE of CBEC can establish customer trust (benevolence, integrity, and ability). Potential parallel multiple mediation effects of customer trust are analyzed, and the relative importance of the three specific mediation paths are compared to answer the research question: “for sustainable development of CBEC, is competence-based influencing more important than good-character influencing”. (2) The moderated mediation effect is tested on the hypothesis model to investigate the potential moderated mechanism in generating consumer online purchasing intention, thus determining the “information haze”. (3) This study analyzes the long-term mechanism of TELE on online purchasing intention and the corresponding optimization strategies for CBEC platforms. Thus making theoretical and management contributions for sustainable development of CBEC platforms.

## Theoretical Background and Hypothesis

### Theoretical Background

The concept of presence was first developed in communication and has gradually expanded into distance learning, human-computer interaction, and marketing (Klein, 2003; Faiola et al., 2013; Baek et al., 2020) with the rise and popularity of the internet. To date, there is no consensus definition; scholars commonly define the connotation and dimensions according to specific research contexts. In general, presence refers to the individual's sense of reality obtained in specific virtual circumstances, and people can experience virtual reality in an artificially constructed environment (Klein, 2003; Baek et al., 2020).

Due to the different classifications, presence is characterized by diversity and complexity, resulting in various research variables, such as product presence, spatial presence, virtual presence, subjective presence, and objective presence. Ijsselsteijn et al. (2000) divided presence into physical and social from the perspective of user cognition, with spatial presence emphasizing “being there” and social presence reflecting “being with others”. In recent years, studies on TELE related to e-commerce have gradually increased. Scholars have focused on the impact mechanism of TELE on consumer attitude and behavior (Mollen and Wilson, 2010; Baek et al., 2020). Various measures and corresponding scales have been developed according to specific research subjects and contexts, such as subjective measures (Shen and Khalifa, 2008) and physiological arousal measures (Baumgartner et al., 2006).

Telepresence was first proposed by Minsky (1980), reflecting the characteristics of virtual TELE (i.e., a sense of being there). Specifically, it refers to a sense of transportation created through a new technique, perceiving the feeling of “being there” rather than the real “being there” (Faiola et al., 2013). In the CBEC context, TELE refers to the operator implanting rich consumption information and using humanoid technology to immerse customers in the virtual simulative scene created by online platforms and retailers. The experience of CBEC consumers during online shopping is similar to offline business transactions in the country of origin. Namely, a sense of being in the country or location being depicted in the pictures (Baek et al., 2020). As a result, the physical distance between consumers and retailers is diminished by an elaborately designed virtual shopping scenario. Lu et al. (2016) confirmed that presence is an effective business strategy, given that it can narrow the psychological distance between consumers and retailers, thereby improving customer relationship quality.

### Hypothesis

#### Mediation Effects of Customer Trust

Trust is generally defined as confidence in the honesty and reliability between business partners (Morgan and Hunt, 1994; Zheng et al., 2017). Nowadays, it has been extended to confidence in fulfilling capability, goodwill, and benevolence of the retailers (Gefen, 2002; Bilgihan, 2016). Gefen (2002) further provided a multidimensional construct containing benevolence, integrity, and ability. In this article, customer trust is defined as the sense of identification and reliability resulting from buyers' satisfaction with the integrity, benevolence, and ability of CBEC platforms and affiliated retailers (Gefen and Straub, 2004). Benevolence trust (**BT**) refers to the buyer's belief that platforms and retailers care about customer interests, treat customers beautifully, and regard consumer interests as their duties (Lu et al., 2016). Integrity trust (**IT**) is the perception that CBEC platforms and retailers uphold honesty as part of their business philosophy and keep their promises (Hwang and Kim, 2007). Ability trust (**AT**) refers to the buyer's belief that the platform and retailers are well versed in product knowledge and can provide customers with the best bargains, related service, and professional guidance (Xu et al., 2021) while excelling in their duties.

Previous studies have widely confirmed the significant relationship between presence and online consumer attitude and behavior (Ou et al., 2014; Baek et al., 2020). For example, Gefen and Straub (2004) found that the presence created by e-commerce platforms can effectively enhance customer trust and activate consumer online purchasing intention. Ou et al. (2014) concluded that TELE promotes BT, IT, and AT and positively influences consumer repurchasing intentions. Similarly, Suntornpithug and Khamalah (2010) found that spatial presence and social presence positively affect online purchasing intention through customer trust. These suggest that TELE strengthens the social connectedness between buyers and sellers by creating an immersive feel during consumption, narrowing the psychological distance between them, and reducing uncertainties in the shopping journey (Mou et al., 2020a). By inference, consumers gradually generate trust in CBEC platforms and retailers and are inclined to implement online purchasing behavior (Lu et al., 2016). Based on these arguments, this article hypothesizes that:

H1: TELE positively affects online purchasing intention through BT (H1a), IT (H1b), and AT (H1c).

#### Moderated Mediation Effects

In CBEC, product information contains three dimensions: product description (**PDE**), product display (**PDI**), and product content (**PC**) (Zhu et al., 2020). PDE refers to the situation where an online product is introduced to target consumers in plain language to meet their concerns and form an overall impression and cognition (e.g., objectivity, understandability, and credibility toward the product) (Park et al., 2007). In this article, PDI is defined as product promotion, which is coupled with network and new techniques (e.g., virtual simulation and augmented reality) in the virtual consumption scenario to generate perceptions, such as impressiveness, attractiveness, and noticeability among potential consumers (Zhu et al., 2020). PC refers to the information related to product attributes that are necessary and informative and affect the consumers' overall assessment of benefits (Horstmann, 2017).

In the pre-purchase stage, online consumers cannot easily discriminate between low and high quality when facing the virtual consumption scene on a cross-border platform (Yeh et al., 2012). The consumers' consumption decisions are full of uncertainties, which include pre-contractual and post-contractual uncertainties (Mou et al., 2020a). Consequently, cross-border platforms and retailers enhance customer trust by presenting detailed product information (Zhu et al., 2019, 2020). Kim et al. (2008) found that quantity and quality can strengthen the credibility and accuracy of product information. Zhu et al. (2019) argue that the length and depth of information communication can reduce uncertainty in consumer decisions, generating greater trust between buyers and sellers. This suggests that when the product information (description, display, and content) is high, a high-quality PDE, a vivid PDI, and an expansive PC can help enhance the psychological experience from TELE (Kronrod and Danziger, 2013; Zhu et al., 2020) and trigger positive consumer association and imagination of the product use effects (Zhu et al., 2020). These would then produce perceived trust (benevolence, integrity, and ability) more easily and have a stronger impact on online purchasing intentions. According to the cue utilization theory, the richer the product information, the more it arouses consumer memory on target products (Krishna, 2012) and mitigates risk perception (Baek et al., 2020). These would help win the trust of online customers and have a cumulative effect on purchasing intentions on cross-border platforms.

In contrast, low product information (PDE, PDI, and PC) leads to poor access to product knowledge and shared shopping experiences, causing information asymmetry (Zhu et al., 2020) and weakening the influence of TELE on consumer behavior. Uncertainties in consumption decisions would increase due to lack of product information (Mou et al., 2020a), deteriorating perceived consumer effectiveness (Troise et al., 2021) and the TELE effects on customer trust (benevolence, integrity, and ability), and weakening the influence on consumer behavior. Accordingly, the following hypotheses are proposed:

H2. The mediation effect of customer trust (BT, H2a; IT, H2b; AT, H2c) is positively moderated by PDE. The higher the PDE, the greater the positive mediation effect of customer trust.

H3. The mediation effect of customer trust (BT, H3a; IT, H3b; AT, H3c) is positively moderated by PDI. The higher the PDI, the greater the positive mediation effect of customer trust.

H4. The mediation effect of customer trust (BT, H4a; IT, H4b; AT, H4c) is positively moderated by PC. The higher the PC, the greater the positive mediation effect of customer trust.

**Figure 1:**
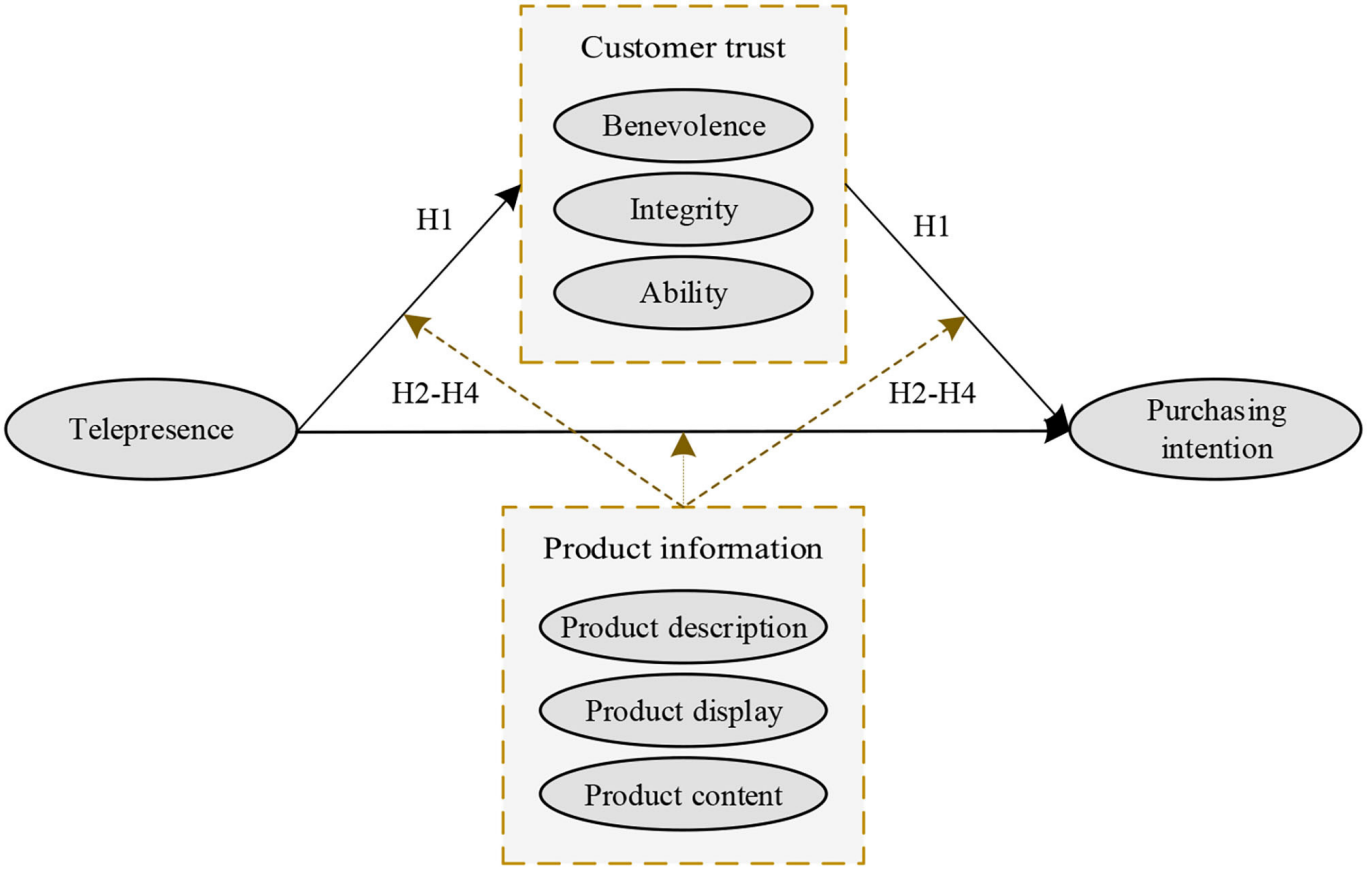
Research model of this study.

According to the theoretical background and hypothesis development, the research model proposed in this study is shown in Figure 1.

## Methodology

### Measurements Design

The measures used in this study had been previously validated in prior studies, with appropriate modifications introduced to fit the context of CBEC and Chinese respondents (Zheng et al., 2017). TELE was adapted from Baek et al. (2020), which is a unidimensional scale containing four items (Cronbach's alpha = 0.887). The revised items are as follows: (1) It makes me imagine I am actually shopping in the product's country of origin; (2) It gives me as much sensory information as I would experience shopping in the country of origin; (3) It provides an experience similar to the one I had when shopping in the country of origin; (4) It gives me a similar experience of purchasing a product in the country of origin. Three-dimensional measures of customer trust were derived from Zhu et al. (2019). Three items were used to measure BT (Cronbach's alpha = 0.842), four items for IT (Cronbach's alpha = 0.903), and four items for AT (Cronbach's alpha = 0.869). The unidimensional scale of purchasing intention (Cronbach's alpha = 0.856) consisted of three items based on Zhu et al. (2019). The three-dimensional scale of production information consisted of 10 items based on Zhu et al. (2020): four measured PDE (Cronbach's alpha = 0.821), three for PDI (Cronbach's alpha = 0.81), and three for PC (Cronbach's alpha = 0.846). All measurement items used a seven-point Likert scale ranging from 1 = strongly disagree to 7 = strongly agree.

All scales used in the study are related to CBEC. These were translated in English by two PhDs and one MS and then back-translated to Chinese to ensure that the items were consistent with the original scale. The initial questionnaire was pilot tested on 30 college students to determine potential errors and evaluate its suitability for the CBEC context. Based on the pilot test results, the final questionnaire was formed. To ensure that the respondents have a high degree of familiarity with CBEC and filter out invalid responses, two questions were placed in the questionnaires. The respondents were asked to fill in the name of their most frequently browsed (or used) CBEC platform, as well as the number of times they have used the platform in the past three months.

### Procedure and Sampling

The formal survey was conducted from September to November 2021 on the Tencent Questionnaire platform, and only the respondents familiar with CBEC transactions were regarded as qualified respondents. Meanwhile, this study selected well-known B2C CBEC platforms in China, such as Amazon, LightInTheBox, and Tmall Global. Particularly, the evaluation objects of all items are the CBEC platform's name filled in by the corresponding respondents due to consumer perceived trust in sellers will transfer to the affiliated platform. The sources of the samples are as follows: (1) classmates and friends of task group members; (2) colleagues; (3) graduate students of task group members and their colleagues. To ensure that the respondents meet the requirements of our study, the screening criteria for invalid samples are as follows: (1) no variation in respondents' answers to all items; (2) The platform name filled in by the respondents is not a mainstream CBEC platform; (3) The respondents had no experience of browsing or shopping on CBEC platforms in the past 3 months. Eventually, 501 respondents participated in the survey, 406 of which were usable samples, with an effective rate of 81.04%. The respondents' characteristics are summarized in Table 1.

**Table 1:** Sample demographic profile (*N* = 406).

**Demographics**	**Category**	**Frequency**	**Percentage (%)**
Gender	Female	204	50.25
	Male	202	49.75
Marital status	Unmarried	233	57.39
	Married	151	37.19
	Others	22	5.42
Age	≤ 30	207	50.99
	31–40	154	37.93
	41–50	34	8.37
	51–60	10	2.46
	≥ 61	1	0.25
Education level	High school and below	13	3.20
	College degree	232	57.14
	Bachelor's degree	116	28.57
	Masters' or above	45	11.08
Monthly income (CNY)	<3,000	176	43.35
	3,000–6,000	78	19.21
	6,001–9,000	101	24.88
	9,000–12,000	34	8.37
	>12,000	17	4.19

### Research Methods

In this study, SPSS 25 was employed for the reliability test and calculate the common method variance (CMV) through exploratory factor analysis, and confirmatory factor analysis was performed in AMOS 23. Moreover, the multiple mediation effect and moderated mediation model test were conducted with multiple regression analysis in the PROCESS v3.3.

## Data Analysis and Results

### Reliability and Validity Test

To examine the reliability of variables, SPSS 25 was used. The results show that Cronbach's alpha coefficient for each construct exceeded the recommended value of 0.7. This suggests that the scales are reliable (Hair et al., 2020), indicating good internal consistency of each scale.

The measurement model was then created in AMOS 23, which included seven latent variables. Confirmatory factor analysis (CFA) was performed to test the validity and composite reliability (CR) (Zheng et al., 2017). The goodness of fit index was subjected to the recommended criterion (recommended criteria as: χ^2^/df < 5, RMSEA < 0.1, CFI > 0.9, GFI > 0.9, TLI > 0.9, IFI > 0.9, NFI > 0.9, and SRMR < 0.08) (Yang et al., 2021). The results are as follows: χ^2^/*df* (767.019/322) = 2.382, RMSEA = 0.058, *GFI* = 0.879, *CFI* = 0.943, *NFI* = 0.906, *TLI* = 0.933, *IFI* = 0.943, and *SRMR* = 0.042. The results show that standardized factor loadings for all items were above 0.5, ranging from 0.571 to 0.872 (*p* < 0.001) and exceeding the recommended threshold of 0.5 (Hair et al., 2020). The AVE values for all latent variables were greater than 0.5 (Hair et al., 2020), and the composite reliability exceeded the recommended 0.7 threshold value, indicating good convergent validity for all scales.

Table 2 shows the results of the discriminant validity test. For each construct, the diagonal arithmetic square root of the average value was greater than its correlations with the other constructs (Fornell and Larcker, 1981). This suggests that the model has good discriminant validity. A nested model comparison was also conducted to further test discriminant validity (Anderson and Gerbing, 1988). The results show that the chi-square difference (see the model comparison) between the restricted model and the default model is significant (Yang et al., 2020). The results are summarized in Table 3.

**Table 2:** Descriptive statistics, correlation matrix and discriminant validity (*N* = 406).

**Variable**	**TELE**	**BT**	**IT**	**AT**	**PI**	**PDE**	**PDI**	**PC**
TELE	**0.814**							
BT	0.647^**^	**0.799**						
IT	0.693^**^	0.758^**^	**0.837**					
AT	0.644^**^	0.728^**^	0.735^**^	**0.792**				
PI	0.567^**^	0.632^**^	0.618^**^	0.716^**^	**0.821**			
PDE	0.410^**^	0.469^**^	0.500^**^	0.473^**^	0.452^**^	**0.742**		
PDI	0.394^**^	0.459^**^	0.452^**^	0.440^**^	0.386^**^	0.620^**^	**0.771**	
PC	0.415^**^	0.512^**^	0.495^**^	0.581^**^	0.495^**^	0.605^**^	0.666^**^	**0.804**
CR	0.887	0.842	0.903	0.870	0.861	0.826	0.815	0.846
Mean	4.532	4.622	4.634	4.825	4.895	4.621	4.712	5.013
S.D.	1.156	1.166	1.164	1.114	1.104	1.058	1.116	1.183

^*^indicates p < 0.05,

** *indicates p < 0.01; the diagonal is the arithmetic square root of the AVE value*.

*TELE, Telepresence; BT, Benevolence trust; IT, Integrity trust; AT, Ability trust; PDE, Product description; PDI, Product display; PC, Product content; PI, Purchasing intention; CR, Composite reliability; SD, Standard deviation*.

*The diagonal is the arithmetic square root of the AVE value (bold values)*.

**Table 3:** Results for further discriminant validity test (*N* = 406).

**Relationships**			**Restricted model**		**Default model**		**Model comparison**		
			**CMIN**	**DF**	**CMIN**	**DF**	**CMIN**	**DF**	**P**
BT	<->	IT	837.253	323	767.019	322	70.233	1	0.000
IT	<->	AT	838.731	323	767.019	322	71.712	1	0.000
IT	<->	PI	1,025.898	323	767.019	322	258.879	1	0.000
IT	<->	PDE	1,136.004	323	767.019	322	368.985	1	0.000
IT	<->	PDI	1,046.217	323	767.019	322	279.197	1	0.000
TELE	<->	IT	1,017.557	323	767.019	322	250.537	1	0.000
IT	<->	PC	1,096.470	323	767.019	322	329.451	1	0.000
BT	<->	PTA	841.565	323	767.019	322	74.546	1	0.000
BT	<->	PI	927.912	323	767.019	322	160.892	1	0.000
BT	<->	PDE	1,072.103	323	767.019	322	305.083	1	0.000
BT	<->	PDI	1,011.005	323	767.019	322	243.986	1	0.000
TELE	<->	BT	934.660	323	767.019	322	167.641	1	0.000
BT	<->	PC	1,018.629	323	767.019	322	251.610	1	0.000
AT	<->	PI	879.791	323	767.019	322	112.772	1	0.000
AT	<->	PDE	1,127.717	323	767.019	322	360.697	1	0.000
AT	<->	PDI	1,043.718	323	767.019	322	276.699	1	0.000
TELE	<->	AT	1,024.265	323	767.019	322	257.245	1	0.000
AT	<->	PC	993.093	323	767.019	322	226.074	1	0.000
PI	<->	PDE	1,132.995	323	767.019	322	365.976	1	0.000
PI	<->	PDI	1,075.220	323	767.019	322	308.201	1	0.000
TELE	<->	PI	1,057.966	323	767.019	322	290.947	1	0.000
PI	<->	PC	1,070.012	323	767.019	322	302.993	1	0.000
PDE	<->	PDI	898.733	323	767.019	322	131.714	1	0.000
TELE	<->	PDE	1,199.698	323	767.019	322	432.679	1	0.000
PDE	<->	PC	937.652	323	767.019	322	170.633	1	0.000
TELE	<->	PDI	1,071.113	323	767.019	322	304.093	1	0.000
PDI	<->	PC	851.073	323	767.019	322	84.054	1	0.000
TELE	<->	PC	1,142.862	323	767.019	322	375.843	1	0.000

*TELE, Telepresence; BT, Benevolence trust; IT, Integrity trust; AT, Ability trust; PDE, Product description; PDI, Product display; PC, Product content; PI, Purchasing intention; CR, Composite reliability; SD, Standard deviation*.

### Common Method Variance (CMV)

The potential threat of CMV may occur in the self-administered survey (Podsakoff et al., 2003). In this study, important steps were taken to address the CMV, including changing the order of the questionnaire items, ensuring the anonymity of the survey, and hiding our research goals. Harman's one-factor test was used to check for CMV. The results show that the first factor explained approximately 45% of the total variance, which is less than 50% percent of the total variance. This suggests that CMV was not a serious threat in the current study.

### Descriptive Statistics

The mean, *SD*, average values, and Pearson's correlations among the constructs are summarized in Table 2. The correlation analysis shows that TELE had a positive correlation with BT (*r* = 0.647, *p* <0.01), IT (*r* = 0.693, *p* <0.01), and AT (*r* = 0.644, *p* <0.01). These preliminary results indicate that TELE had comparable activation powers on BT, IT, and AT. BT (*r* = 0.632, *p* < 0.01), IT (*r* = 0.618, *p* < 0.01), and AT (*r* = 0.716, *p* < 0.01) were all positively correlated with online purchasing intention, while the moderated variables had low or moderate correlation with the remaining variables.

### Hypothesis Test

#### Parallel Multiple Mediation Effect Test

To eliminate multicollinearity which is reflected in the correlation analysis, the mean structure of the latent variable was adopted in the hypothesis testing. The mediation test process recommended by Zhao et al. (2010) and the test method proposed by Hayes (2018) was used to test potential parallel multiple mediation effects. In the bootstrapping algorithm, sampling was set to 5,000 in PROCESS v3.3, and the confidence level was set to 95%. Model 4 was selected to perform the mediation effect test. The results in Table 4 show that the total indirect effect was significant (BootLLCI = 0.3247 and BootULCI = 0.5157), with the value equal to 0.4184. The specific indirect effects of BT (BootLLCI = 0.0391 and BootULCI = 0.2161) and AT (BootLLCI = 0.2103 and BootULCI = 0.419) were 0.1217 and 0.3146, respectively, and were significant. For IT, the specific indirect effect was insignificant (BootLLCI = −0.1386 and BootULCI = 0.1024), and the point estimation was −0.018.

**Table 4:** The total indirect effects and specific indirect effects (*N* = 406).

**Mediation effect**	**Paths**	**Effect**	**Boot SE**	**Boot LLCI**	**Boot ULCI**
Total indirect effect	TELE → Trust → PI	0.4148	0.0491	0.3247	0.5157
	TELE → BT → PI	0.1217	0.0461	0.0391	0.2161
Specific indirect effect	TELE → IT → PI	−0.0180	0.0613	−0.1386	0.1024
	TELE → AT → PI	0.3146	0.0524	0.2103	0.4190
Differences	Mediation effect (BT-IT)	0.1397	0.0900	−0.0320	0.3172
	Mediation effect (BT-AT)	−0.1929	0.0778	−0.3423	−0.0402
	Mediation effect (IT-AT)	−0.3326	0.0966	−0.5214	−0.1430

*TELE, Telepresence; BT, Benevolence trust; IT, Integrity trust; AT, Ability trust; PI, Purchasing intention; CR, Composite reliability; SD, Standard deviation; SE, Standard error; LLCI, Lower limit confidence level; ULCI, Upper limit confidence level*.

In addition, after comparing the sizes of three specific mediation paths, the mediation effect of AT was calculated to be significantly stronger than that of BT and IT. The differences in mediation effect were −0.1929 (BootLLCI = −0.3423 and BootULCI = −0.0402) and −0.3326 (BootLLCI = −0.5214 and BootULCI = −0.143), respectively. Taken together, the results indicate that H1, H1a, and H1c are confirmed, while H1b is rejected.

The results of the mediation model detailing the relationships among the variables are presented in Table 5. TELE positively influenced BT (BootLLCI = 0.5716 and BootULCI = 0.7329), IT (BootLLCI = 0.6179 and BootULCI = 0.7725), and AT (BootLLCI = 0.5311 and BootULCI = 0.7086). After controlling for the effect of the independent variable, BT (BootLLCI = 0.0612 and BootULCI = 0.3263) and AT (BootLLCI = 0.3444 and BootULCI = 0.6615) positively affected purchasing intention, while IT had no significant effect on purchasing intention (BootLLCI = −0.1962 and BootULCI = 0.1474). These findings indicate that the effects of TELE on BT, IT, and AT were not significantly different. and the influence of AT on purchasing intention was significantly greater than that of BT.

**Table 5:** The results for path coefficients (*N* = 406).

**Paths**	**Standardized coefficient**	**Unstandardized coefficient**	**Boot SE**	**Boot LLCI**	**Boot ULCI**
TELE → BT	0.6468	0.6521	0.0414	0.5716	0.7329
TELE → IT	0.6926	0.6976	0.0389	0.6179	0.7725
TELE → AT	0.6442	0.6205	0.0454	0.5311	0.7086
BT → PI	0.1970	0.1867	0.0696	0.0612	0.3263
IT → PI	−0.0272	−0.0258	0.0876	−0.1962	0.1474
AT → PI	0.5113	0.5070	0.0812	0.3444	0.6615

*TELE, Telepresence; BT, Benevolence trust; IT, Integrity trust; AT, Ability trust; PI, Purchasing intention; SE, Standard error; LLCI, Lower limit confidence level; ULCI, Upper limit confidence level*.

#### Moderated Mediation Effects Test

The moderated mediation effects test approach proposed by Hayes (2018) was used in this study. Using PROCESS v3.3, the Bootstrapping samples were set to 5,000, and the confidence level was set to 95%. Model 59 was selected for testing the moderated mediation models, also known as the conditional process model (Hayes, 2018). According to Hayes (2018), the differences in the significance of the conditional indirect effects at specific levels of the moderator (i.e., mean ± 1SD) would support the moderated mediation model. The results presented in Table 6 indicate that the indirect effect of BT was significant for low PDE (B = 0.1529; BootLLCI = 0.0537 and BootULCI = 0.2486), but not significant for high PDE (B = 0.049; BootLLCI = −0.0076 and BootULCI = 0.1602). In addition, the indirect effect of IT was insignificant for consumers with low PDE levels (B = −0.0882; BootLLCI = −0.2289 and BootULCI = 0.0558), as well as those with high PDE levels (B = 0.0226; BootLLCI = −0.12 and BootULCI = 0.1374). The indirect effect of AT was significant for consumers with low levels of PDE (B = 0.2901; BootLLCI = 0.1536 and BootULCI = 0.4444) and those experiencing high PDE levels (B = 0.2254; BootLLCI = 0.121 and BootULCI = 0.3367). The pairwise contrasts between conditional indirect effects were then analyzed, the findings further indicate no significant conditional indirect effects (mediator = IT and AT). Taken together, the results suggest that the indirect effect of TELE on purchasing intention through BT was negatively moderated by PDE. Hence, H2 and H2a are partially confirmed, while H2b and H2c are rejected.

**Table 6:** Results for the moderated mediation effects (*N* = 406).

**Moderator**	**Mediator**	**Mean, Mean ± 1SD**	**Effect**	**Boot SE**	**Boot LLCI**	**Boot ULCI**
PDE	BT	−1.0583	0.1529	0.0500	0.0537	0.2486
		0.0000	0.0985	0.0332	0.0376	0.1691
		1.0583	0.0490	0.0428	−0.0076	0.1602
	IT	−1.0583	−0.0882	0.0731	−0.2289	0.0558
		0.0000	−0.0286	0.0526	−0.1345	0.0682
		1.0583	0.0226	0.0652	−0.1200	0.1374
	AT	−1.0583	0.2901	0.0736	0.1536	0.4444
		0.0000	0.2582	0.0463	0.1698	0.3508
		1.0583	0.2254	0.0552	0.1210	0.3367
		Mean, Mean ± 1SD	Effect	Boot SE	Boot LLCI	Boot ULCI
PDI	BT	−1.1160	0.1621	0.0443	0.0776	0.2532
		0.0000	0.9990	0.0346	0.0428	0.1790
		1.1160	0.0413	0.0500	−0.0189	0.1720
	IT	−1.1160	−0.0326	0.0667	−0.1639	0.0979
		0.0000	−0.0183	0.0537	−0.1304	0.0852
		1.1160	−0.0031	0.0810	−0.1876	0.1327
	AT	−1.1160	0.2817	0.0669	0.1628	0.4224
		0.0000	0.2713	0.0468	0.1834	0.3664
		1.1160	0.2606	0.0641	0.1439	0.3942
		Mean, Mean ± 1SD	Effect	Boot SE	Boot LLCI	Boot ULCI
PC	BT	−1.1835	0.1290	0.0472	0.0338	0.2180
		0.0000	0.0928	0.0343	0.0353	0.1667
		1.1835	0.0596	0.0531	−0.0128	0.1897
	IT	−1.1835	0.0590	0.0822	−0.0959	0.2281
		0.0000	−0.0025	0.0494	−0.1035	0.0939
		1.1835	−0.0639	0.0721	−0.2102	0.0721
	AT	−1.1835	0.1753	0.0666	0.0599	0.3253
		0.0000	0.2196	0.0419	0.1366	0.3020
		1.1835	0.2568	0.0560	0.1374	0.3561

*TELE, Telepresence; BT, Benevolence trust; IT, Integrity trust; AT, Ability trust; PI, Purchasing intention; PDE, Product description; PDI, Product display; PC, Product content; SE, Standard error; LLCI, Lower limit confidence level; ULCI, Upper limit confidence level*.

Similarly, the indirect effect of BT was significant for low PDI (B = 0.1621; BootLLCI = 0.0776 and BootULCI = 0.2532), but not significant for high PDI (B = 0.0413; BootLLCI = −0.0189 and BootULCI = 0.172). The indirect effect of IT was insignificant for consumers with low PDI levels (B = −0.0326; BootLLCI = −0.1639 and BootULCI = 0.0979), as well as those with high PDI levels (B = −0.0031; BootLLCI = −0.1876 and BootULCI = 0.1327). For AT, the indirect effect was significant for consumers with low PDI levels, (B = 0.2817; BootLLCI = 0.1628 and BootULCI = 0.4224) and those with high levels of PDI (B = 0.2606; BootLLCI = 0.1439 and BootULCI = 0.3942). Furthermore, the pairwise contrasts between conditional indirect effects indicate no significant conditional indirect effects (mediator = IT and AT). The results suggest that the indirect effect of TELE on purchasing intention through BT is negatively moderated by PDI. Hence, H3 and H3a are partially confirmed, while H3b and H3c are rejected.

Finally, the indirect effect of BT was significant for low PC conditions (B = 0.129; BootLLCI = 0.0338 and BootULCI = 0.218), but not significant for those with high PC (B = 0.0596; BootLLCI = −0.0128 and BootULCI = 0.1897). The indirect effect of IT was not significant for consumers with low PC levels (B = 0.059; BootLLCI = −0.0959 and BootULCI = 0.2281), as well as those with high levels of PC (B = −0.0639; BootLLCI = −0.2102 and BootULCI = 0.0721). For AT, its indirect effect was significant for consumers experiencing low levels of PC (B = 0.1753; BootLLCI = 0.0599 and BootULCI = 0.3253) and those with high levels (B = 0.2568; BootLLCI = 0.1374 and BootULCI = 0.3561). The pairwise contrasts between conditional indirect effects indicate no significant conditional indirect effects (mediator = IT and AT). The results suggest that the indirect effect of TELE on purchasing intention through BT is negatively moderated by PC. Hence, H4 and H4a are partially confirmed, while H4b and H4c are rejected.

**Figure 2:**
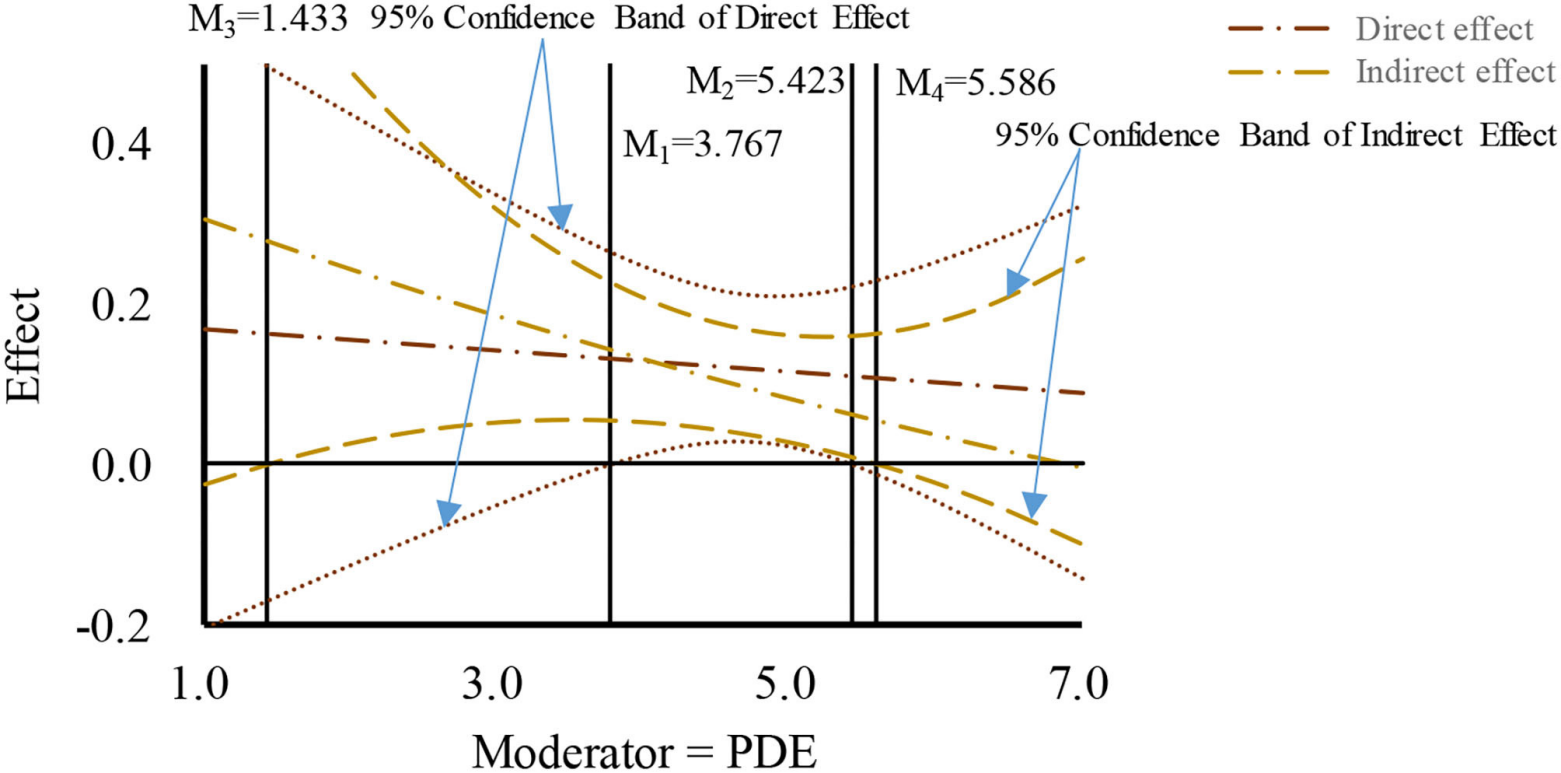
Moderated mediation effect of product description.

**Figure 3:**
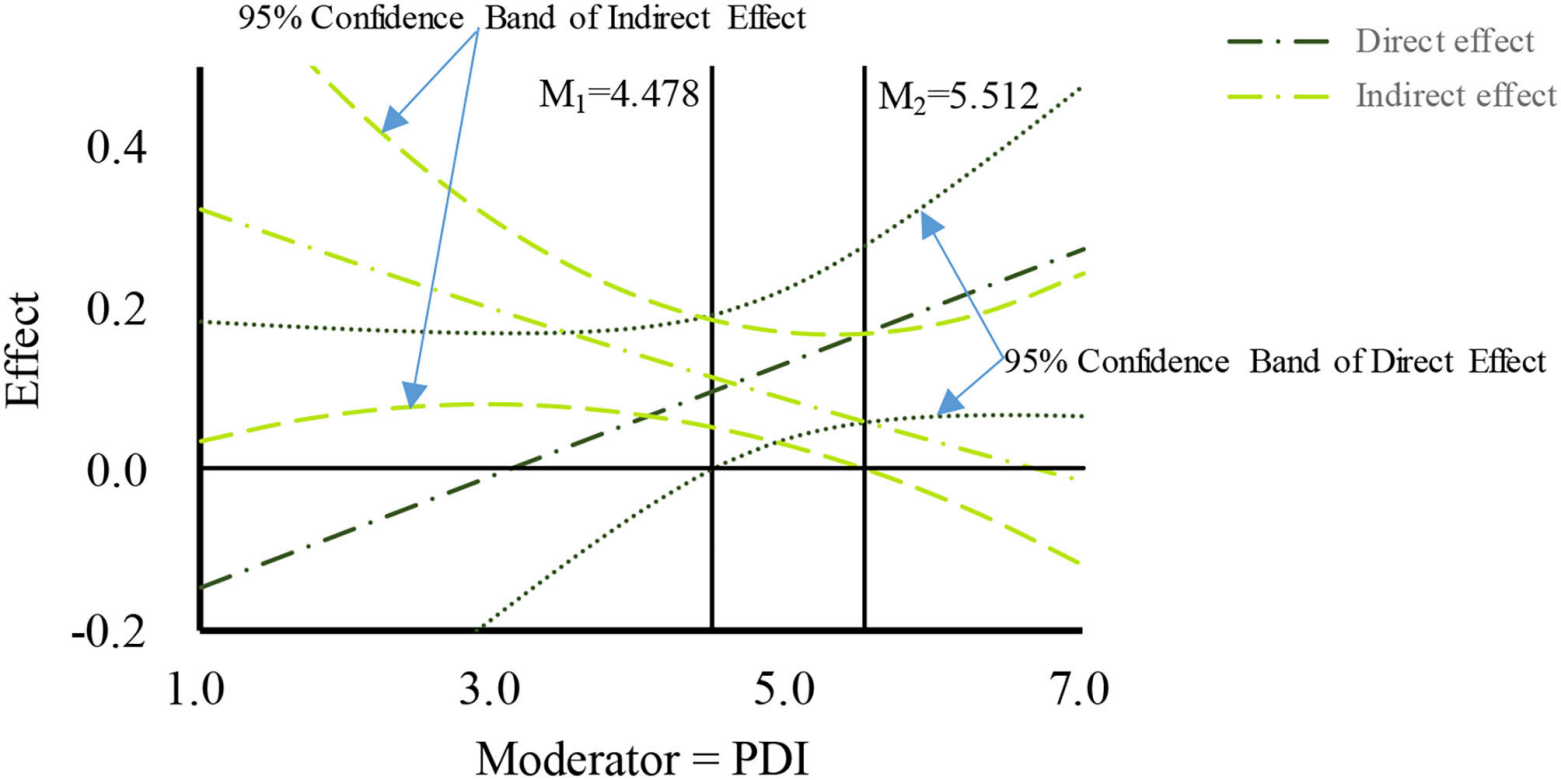
Moderated mediation effect of product display.

**Figure 4:**
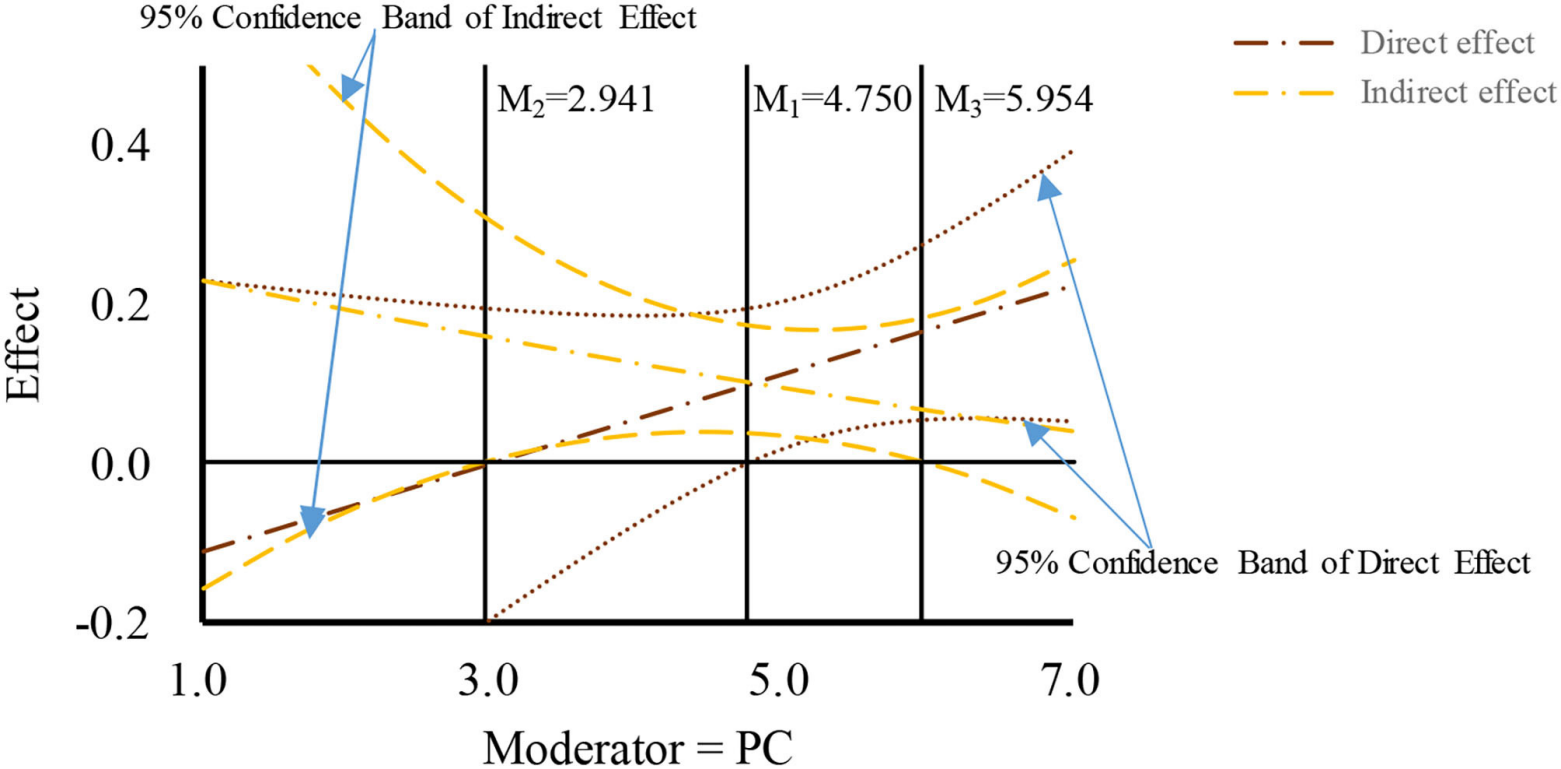
Moderated mediation effect of product content.

Based on the drawing method proposed by Hayes (2018) and Preacher et al. (2007), the moderated mediation effects were plotted using the moderated effect when the moderator value is equivalent to mean and mean ± 1SD, respectively, and the corresponding confidence intervals. The concrete number value of the region of significance and the 95% confidence band were determined using the Johnson-Neyman (J-N) method. As shown in Figures 2–4, the PDE, PDI, and PC were found to negatively moderate the mediation path of TELE on purchasing intention through BT. Concretely, The indirect effect of TELE on purchasing intention through BT was found to be significant when the raw value of moderator (PDE) was between 1.433 and 5.586 (1 ≤ PDE ≤ 7), when the raw value of moderator (PDI) was between 1 and 5.512 (1 ≤ PDE ≤ 7), or when the raw value of moderator (PC) was between 2.941 to 5.954 (1 ≤ PC ≤ 7). Moreover, The figures also present the raw value ranges of the three moderators when the direct effects were significant.

## Discussion, Implication and Limitations

### Discussion of the Results

This article is one of the first attempts to determine the influencing mechanism of TELE on consumer purchasing intention through customer trust in a CBEC context, aiming to find an effective path to improve purchasing intention. Potential “information haze” was also investigated, forming the following findings.

First, TELE created by CBEC can effectively enhance customer trust, thus determining the effective route for long-term success. In line with previous studies (e.g., Baek et al., 2020), TELE was found to play the role of “virtual-direct experience”, successfully activating the consumer's BT, IT, and AT (Zhu et al., 2019). These results support the hypothesis that TELE increases persuasion on online consumers without having to experience it physically by generating positive beliefs toward the products and retailers (Klein, 2003). Another important finding is that TELE has similar activation power on BT, IT, and AT. This finding may partly be explained by Li et al. (2002), suggesting that TELE enables consumers to make an accurate judgment and form appropriate cognition toward products without being in a real-world scenario, thus comprehensively evaluating the consumption value. These findings demonstrate that the continuous improvement of BT, IT, and AT can result from the gradual accumulation of the consumer's positive affective (Hwang and Kim, 2007). Consequently, our findings validate previous conclusions (e.g., Baek et al., 2020; Mou et al., 2020a) that the long-term success of TELE depends on increasing customer trust rather than stimulating impulse purchases (Zhu et al., 2020).

Second, compared with BT and IT (good character), AT (ability orientation) has a stronger impact on purchasing intention, which is the core power driving sales growth. Using TELE to guide and shape AT is the most effective means to expand online consumption. While this finding is contrary to that of Knoll and Gill (2011), it supports the conclusions of Schlosser et al. (2006), Lu et al. (2016), and Wu and Tsang (2008), which found that AT is the most important driving factor affecting online purchasing intention. The diverging results in previous studies may be due to the differences in how BT, IT, and AT affect online purchasing intention caused by varying objects and contexts. This preliminary assessment also suggests that the influence of three-dimensional customer trust on the purchasing preference of offline customers may vary significantly and differ from the results in this study. Investigating the multiple mediation effect of customer trust extends the analyses of Zhu et al. (2019) and Hwang and Lee (2012) and offers new perspectives on the driving mechanism of TELE on online purchasing intention in CBEC. The results indicate that shaping AT and BT can influence online purchasing intention effectively. However, contrary to expectations, IT did not play a significant mediating role. While these results are in agreement with Wu and Tsang's (2008), they are contrary to Hwang and Lee's (2012) study, possibly due to differences in research contexts.

Third, the most interesting finding is that the “information haze” may occur in CBEC due to information overload. The moderated mediation mechanism in this study provides a greater understanding of the moderated effects of product information. The results indicate that product information overload can easily bring CBEC consumers to decision dilemmas. These findings build on the research of Zhu et al. (2019), Zhu et al. (2020), and Mou et al. (2020a). Thus, it can be concluded that enriching product information by the sellers alone would be insufficient to enhance the mediation effect of customer trust. It is sometimes more important to optimize information combination, choose appropriate information transmitting media, and improve information processing and information extracting fluency.

### Implications

#### Theoretical Implications

The findings contribute to the theory and address the current knowledge gaps in several ways. First, by establishing the long-term effects of TELE on consumer purchasing intention in the CBEC context, this article overcomes the shortcomings of subjectively measuring consumer repurchasing behavior and focuses on the internal impetus of consumer behavior (i.e., customer trust) to determine the specific and effective realizing routes. This new understanding helps improve the theoretical direction for the application of TELE in the field of CBEC.

In addition, this study developed a theoretical framework of the TELE on online purchasing intention with customer trust as a multiple mediation variable, overcoming the shortcomings in prior literature that treat customer trust as a unidimensional construct. In particular, the bridging roles played by BT, IT, and AT were comprehensively analyzed. By examining the multiple mediation effects of customer trust, this study adds to existing knowledge of specific routes for the long-term success of TELE in purchasing intention. The insights gained from this study would help generate new ideas for CBEC operators to optimize their business strategies and provide a theoretical reference for sustainable development. This would address and bridge the literature gaps in customer trust, particularly in CBEC.

Finally, this study is one of the first empirical investigations into the “information haze” phenomenon caused by product information overload. Unlike previous studies that found a positive correlation between product information and purchasing intention and use product information as a predictor, our results suggest that excessive information in the CBEC marketplace may hinder the formation of purchasing intention. Thus, the boundary conditions for product information to play an active role are determined. This study also comprehensively evaluated the moderating roles of the PDE, PDI, and PC on the mediation paths. The findings suggest that consumers in the CBEC marketplace are confronted with multiple complex information combinations in the pre-contractual stage that could affect their purchasing preferences and the following consumption decision. These findings indicate two possibilities in the product information combination faced by consumers: “consistent information impact” and “conflicting information impact”. The results provide theoretical insights for CBEC operators to optimize their information management strategies.

#### Management Implications

The results have important management implications. First, CBEC platforms and affiliated sellers should create a more perceptible virtual consumption scenario and narrow the “physical distance” and “psychological distance” between them and online consumers. They should consider using simulation technology and augmented reality to increase the consumers' sense of “being there” and immersion in virtual space. Vividness and interactiveness should be fully integrated into these new immersive techniques so that the utilitarian and hedonic values delivered by CBEC platforms and sellers are better experienced by online consumers. Furthermore, CBEC operators should encode high fluency signals that can map product quality and convey the ability and benevolence of their e-commerce platforms to narrow the physical and psychological distance between buyer and seller. The most effective strategy to strengthen TELE is by developing and reinforcing interactions, including those between the consumer and the e-commerce platform and between the consumer and seller. Therefore, in the course of interaction, CBEC operators should implement targeted measures, such as creating unique branding and marketing strategies, improving the image and perception of the country of origin, embedding elements of consumer culture in the country of origin, focusing on high-quality products for customer retention, and attracting consumers with featured products.

Cross-border e-commerce operators should also adopt an ability-oriented business philosophy and develop good corporate character. The results suggest that strengthening the abilities of the CBEC platform and seller is the most effective way to expand the influence of TELE on consumer purchasing intention and is an inherent requirement for sustainable development. To this end, practitioners should decompose the components of ability according to the CBEC context and target the aspects that concern and affect potential consumers. Practitioners should be able to delaminate ability and anchor its key components, focusing on core capabilities while also considering the big picture. In addition, practitioners should develop their corporate pro-social character, show benevolence to consumers, and always be mindful of essential consumer interests. Platforms and sellers should adopt a customer-centric philosophy instead of a profit-centric mentality, and reduce short-sighted behavior in order to foment customer loyalty and long-term profitability.

Another important practical implication is that appropriate and matching strategies for product information combination should be selected to minimize information haze. For the information sender, the misconception that the information quantity determines the persuasion effect should be corrected, and the product information quality should be constantly improved to enhance consumer processing fluency, so that avoid the consumer's decision dilemma resulting from information overload. Platforms and sellers should be clear that the amount of information is not always valid, they should focus on providing accurate essential information to generate an information collaboration effect. For the information receiver, CBEC operators should help consumers improve their consumption skills and increase their information reception abilities and cognitive levels. Moreover, operators should consider developing release channels to allow more convenient reception of product information. These measures would support consumers in enriching their consumption knowledge and mitigate decision-making anxieties caused by information dilemmas and uncertainties.

### Limitation and Future Research

Despite the important findings, there are a few limitations that must be addressed. First, the causal inferences were limited by the cross-sectional data. Future studies could further explore the causal relationships in the research model using a longitudinal study. Second, this study did not focus on the moderated effects of the matching degree of positive and negative information combination on the formation of consumer purchasing intention, which could be further analyzed in subsequent research. Finally, the empirical analysis was conducted in the Chinese context, which may limit the generalizability of the research conclusions. Cross-national, multicultural, and cross-time assessments should be considered in succeeding studies.

## Conclusion

This study developed a theoretical model to assess consumer purchasing intention in a CBEC context and determine whether the TELE of CBEC can establish customer trust. The results suggest that TELE has the potential to fulfill long-term success and that ability orientation is the most effective way to improve online consumer preference. Good character and ability orientation of CBEC platforms can co-exist and be mutually enriching. Another important purpose of this study was to determine the potential “information haze”, and the analysis of moderated mediation models undertaken in this study has addressed this goal and extended our knowledge of the negative consequences of information overload on CBEC platforms, and provided new perspectives on how to eliminate uncertainties in online shopping.

## Data Availability Statement

The original contributions presented in the study are included in the article/supplementary material, further inquiries can be directed to the corresponding authors.

## Author Contributions

HG and UR carried out the conceptualization. HG and XY were involved in the methodology. XY and YY conducted the investigation. UR and XY performed the formal analysis. UR supervised the study. XY performed the visualization. HG, UR, and XY validated the manuscript. HG, UR, XY, and YY were involved in writing the original draft preparation and performed the writing, reviewing, and editing. All authors have read and agreed to the published version of the manuscript.

## Funding

This study was supported by Philosophy and Social Science Major Project of Colleges in Jiangsu Province (Grant No. 2020SJZDA082). Special Fund for Outstanding Talented Teachers in Wuxi Vocational Institute of Commerce (Grant Nos. RS20JS01 and RS21TY01).

## Conflict of Interest

The authors declare that the research was conducted in the absence of any commercial or financial relationships that could be construed as a potential conflict of interest.

## Publisher's Note

All claims expressed in this article are solely those of the authors and do not necessarily represent those of their affiliated organizations, or those of the publisher, the editors and the reviewers. Any product that may be evaluated in this article, or claim that may be made by its manufacturer, is not guaranteed or endorsed by the publisher.

## References

[B1] Anderson J. C. Gerbing D. W. (1988). Structural equation modeling in practice: A review and recommended two-step approach. Psychol Bull. 103, 411–423. 10.1037/0033-2909.103.3.411

[B2] Baek E. Lee H. K. Choo H. J. (2020), Cross-border online shopping experiences of Chinese shoppers. Asia Pac J Market Lo. 32, 366–385. 10.1108/APJML-03-2018-0117

[B3] Baumgartner T. Valko L. Esslen M. Jäncke L. (2006). Neural correlate of spatial presence in an arousing and noninteractive virtual reality: An EEG and psychophysiology study. Cyberpsychol Behav. 9, 30–45. 10.1089/cpb.2006.9.30 16497116

[B4] Bilgihan A. (2016). Gen y customer loyalty in online shopping: an integrated model of trust, user experience and branding. Comput Hum Behav. 61, 103–113. 10.1016/j.chb.2016.03.014

[B5] Chen N. Yang Y. (2021). The impact of customer experience on consumer purchase intention in cross-border e-commerce——taking network structural embeddedness as mediator variable. J Retail Consum Serv. 59, 102344. 10.1016/j.jretconser.2020.102344

[B6] Escobar-Rodríguez T. Bonsón-Fernández R. (2017). Analysing online purchase intention in Spain: Fashion e-commerce. Inf Syst E-Bus Manag. 15, 599–622. 10.1007/s10257-016-0319-6

[B7] Faiola A. Newlon C. Pfaff M. Smyslova O. (2013). Correlating the effects of flow and telepresence in virtual worlds: Enhancing our understanding of user behavior in game-based learning. Comput Hum Behav. 29, 1113–1121. 10.1016/j.chb.2012.10.003

[B8] Falk M. Hagsten E. (2015). E-commerce trends and impacts across europe, international. Int J Prod Econ. 170, 357–369. 10.1016/j.ijpe.2015.10.003

[B9] Fornell C. Larcker D. F. (1981). Structural equation models with unobservable variables and measurement error: Algebra and statistics. J Marketing Res. 18, 382–388. 10.1177/002224378101800313

[B10] Gefen D. (2002). Reflections on the dimensions of trust and trustworthiness among online consumers. Data Base Adv Inf Sy. 33, 38–53. 10.1145/569905.569910

[B11] Gefen D. Straub D. (2004). Consumer trust in B2C e-commerce and the importance of social presence: Experiments in e-products and e-services. Omega. 31, 407–424. 10.1016/j.omega.2004.01.006

[B12] Hair J. F. Jr. Black W. C. Babin B. J. Anderson R. E. (2020). Multivariate Data Analysis, 8th Edn. Upper Saddle River, NJ: Prentice Hall.

[B13] Han J. H. Kim H. M. (2019). The role of information technology use for increasing consumer informedness in cross-border electronic commerce: an empirical study. Electron Commer R A. 34, 100826. 10.1016/j.elerap.2019.100826

[B14] Hassanein K. Head M. M. (2007). Manipulating perceived social presence through the web interface and its impact on attitude towards online shopping. Int J Hum-Comput St. 65, 689–708. 10.1016/j.ijhcs.2006.11.018

[B15] Hayes A. F. (2018). Introduction to Mediation, Moderation, and Conditional Process Analysis: A Regression-Based Approach. New York, NY: The Guilford Press.

[B16] Horstmann F. (2017). Measuring the shopper's attitude toward the point of sale display: Scale development and validation. J Retail Consum Serv. 36, 112–123, 10.1016/j.jretconser.2017.01.011

[B17] Hsu M. H. Chang C. M. Chuang L. W. (2015). Understanding the determinants of online repeat purchase intention and moderating role of habit: The case of online group-buying in Taiwan. Int J Inform Manage. 35, 45–56. 10.1016/j.ijinfomgt.2014.09.002

[B18] Hsu M. H. Chuang L. W. Hsu C. S. (2014). Understanding online shopping intention: The roles of four types of trust and their antecedents. Internet Res. 24, 332–352. 10.1108/IntR-01-2013-0007

[B19] Hwang Y. Kim D. J. (2007). Customer self-service systems: the effects of perceived web quality with service contents on enjoyment, anxiety, and e-trust. Decis Support Syst. 43, 746–760. 10.1016/j.dss.2006.12.008

[B20] Hwang Y. Lee K. C. (2012). Investigating the moderating role of uncertainty avoidance cultural values on multidimensional online trust. Inform Manage-Amster. 49, 171–176. 10.1016/j.im.2012.02.003

[B21] Ijsselsteijn W. De Ridder H. Freeman J. Avon S. (2000). Presence: concept, determinants, and measurements, in Proceedings of the SPIE 3959. p.520–529.

[B22] Kim D. J. Ferrin D. L. Rao H. R. (2008). A trust-based consumer decision-making model in electronic commerce: the role of trust, perceived risk, and their antecedents. Decis Support Syst. 44, 544–564. 10.1016/j.dss.2007.07.001

[B23] Klein L. (2003). Creating virtual product experiences: The role of telepresence. J. Interact. Advert. 17, 41–55. 10.1002/dir.10046

[B24] Knoll D. L. Gill H. (2011). Antecedents of trust in supervisors, subordinates, and peers. J. Manage. Psychol. 26, 313–330. 10.1108/02683941111124845

[B25] Krishna A. (2012). An integrative review of sensory marketing: Engaging the senses to affect perception, judgment and behavior. J. Consum. Psychol. 22, 332–351. 10.1016/j.jcps.2011.08.003

[B26] Kronrod A. Danziger S. (2013), Wii will rock you!' the use effect of figurative language in consumer reviews of hedonic utilitarian consumption. J. Consum. Res. 40, 726–739. 10.1086/671998

[B27] Kwak J. Zhang Y. Yu J. (2019). Legitimacy building and e-commerce platform development in China: the experience of Alibaba. Technol Forecast Soc. 139, 115–124, 10.1016/j.techfore.2018.06.038 32287407 PMC7127782

[B28] Li H. Daugherty T. Biocca F. (2002). Impact of 3-D advertising on product knowledge, brand attitude, and purchase intention: The mediating role of presence. J. Advertising. 31, 43–57. 10.1080/00913367.2002.10673675

[B29] Lin T. C. Huang S. L. Hsu C. J. (2015). Dual-factor model of loyalty to IT product–the case of smartphones. Int. J. Inform. Manage. 35, 215–228. 10.1016/j.ijinfomgt.2015.01.001

[B30] Lu B. Fan W. Zhou M. (2016). Social presence, trust, and social commerce purchase intention: an empirical research. Comput. Hum. Behav. 56, 225–237. 10.1016/j.chb.2015.11.057

[B31] Minsky M. (1980). Telepresence. OMNI Magazine. p. 44–52.

[B32] Mollen A. Wilson H. (2010). Engagement, telepresence and interactivity in online consumer experience: reconciling scholastic and managerial perspectives. J. Bus. Res. 63, 919–925. 10.1016/j.jbusres.2009.05.014

[B33] Morgan R. M. Hunt S. D. (1994). The commitment-trust theory of relationship marketing. J. Marketing. 58, 20–38. 10.1177/002224299405800302

[B34] Mou J. Cohen J. Dou Y. Zhang B. (2020a), International buyers' repurchase intentions in a Chinese cross-border e-commerce platform: a valence framework perspective. Internet Res. 30, 403–437. 10.1108/INTR-06-2018-0259

[B35] Mou J. Zhu W. Benyoucef M. (2020b). Impact of product description and involvement on purchase intention in cross-border e-commerce. Ind Manage Data Syst. 120, 567–586. 10.1108/IMDS-05-2019-0280

[B36] Ou C. X. Pavlou P. A. Davison R. M. (2014). Swift guanxi in onlie marketplaces: The role of computer-mediated communication technologies. Mis Quart. 38, 209–230. 10.25300/MISQ/2014/38.1.10

[B37] Park D. H. Lee J. Han I. (2007). The effect of on-line consumer reviews on consumer purchasing intention: The moderating role of involvement. Int J Electron Comm. 11, 125–148. 10.2753/JEC1086-4415110405

[B38] Park J. Lennon S. J. Stoel L. (2005). On-line product presentation: Effects on mood, perceived risk, and purchase intention. Psychol Market. 22, 695–719. 10.1002/mar.20080

[B39] Pavlou P. A. Liang H. Xue Y. (2007). Understanding and mitigating uncertainty in online exchange relationships: a principal-agent perspective. Mis Quart. 31, 105–136. 10.2307/25148783

[B40] Podsakoff P. M. MacKenzie S. B. Lee J. Y. Podsakoff N. P. (2003), Common method biases in behavioral research: a critical review of the literature recommended remedies. J Appl Psychol. 88, 879–903. 10.1037/0021-9010.88.5.879 14516251

[B41] Preacher K. J. Rucker D. D. Hayes A. F. (2007). Addressing moderated mediation hypotheses: Theory, methods, and prescriptions. Multivar Behav Res. 42, 185–227. 10.1080/00273170701341316 26821081

[B42] Qi X. Chan J. H. Hu J. Li Y. (2020). Motivations for selecting cross-border e-commerce as a foreign market entry mode. Ind Market Manag. 89. 50–60, 10.1016/j.indmarman.2020.01.009

[B43] Schlosser A. E. White T. B. Lloyd S. M. (2006). Converting web site visitors into buyers: How web site investment increases consumer trusting beliefs and online purchase intentions. J. Marketing. 70, 133–148. 10.1509/jmkg.70.2.133

[B44] Shen K. N. Khalifa M. (2008). Exploring multidimensional conceptualization of social presence in the context of online communities. Int J Hum-Comput Int. 24, 722–748. 10.1080/10447310802335789

[B45] Strzelecki A. (2019). Key features of e-tailer shops in adaptation to cross-border e-commerce in the EU. Sustainability-Basel. 11, 1589. 10.3390/su11061589

[B46] Suntornpithug N. Khamalah J. (2010). Machine and person interactivity: The driving forces behind influences on consumers' willingness purchase online. J. Electron. Commer. Re. 11, 299–325.

[B47] Troise C. O'Driscoll A. Tani M. Prisco A. (2021). Online food delivery services and behavioural intention – a test of an integrated TAM and TPB framework. Brit Food J. 123, 664–683. 10.1108/BFJ-05-2020-0418

[B48] Wu J-J. Tsang Alex S. L. (2008). Factors affecting members' trust belief and behaviour intention in virtual communities. Behav. Inform Technol. 27, 115–125. 10.1080/01449290600961910

[B49] Xiao L. Guo F. Yu F. Liu S. (2019). The effects of online shopping context cues on consumers' purchase intention for cross-border e-commerce sustainability. Sustainability Basel. 11. 2777. 10.3390/su11102777

[B50] Xu X. Y. Tayyab S. Chang F. K. Zhao K. (2021). Hierarchical value-attainment paths of CBEC consumers: a means-end-chain perspective. Internet Res. 31, 699–736, 10.1108/INTR-10-2019-0397

[B51] Yang X. Chen S. Zhang L. (2020). Promoting sustainable development: A research on residents' green purchasing behavior from a perspective of the goal-framing theory. Sustain. Dev. 28, 1208–1219. 10.1002/sd.2070

[B52] Yang X. Tseng Y. Lee B. (2021). Merging the social influence theory and the goal-framing theory to understand consumers' green purchasing behavior: does the level of sensitivity to climate change really matter? Front. Psychol. 12, 766754. 10.3389/fpsyg.2021.766754 34790155 PMC8591022

[B53] Yeh J. Hsiao K. Yang W. (2012). A study of purchasing behavior in Taiwan's online auction websites: effects of uncertainty and gender differences. Internet. Res. 22, 98–115. 10.1108/10662241211199988

[B54] Zhao X. Lynch J. G. Chen Q. (2010). Reconsidering Baron and Kenny: Myths and truths about mediation analysis. J. Consum. Res. 37, 197–206. 10.1086/651257

[B55] Zheng X. Lee M. Cheung C. M. K. (2017), Examining e-loyalty towards online shopping platforms: the role of coupon proneness value consciousness. Internet. Res. 27. 709–726. 10.1108/IntR-01-2016-0002

[B56] Zhu W. Mou J. Benyoucef M. (2019). Exploring purchase intention in cross-border e-commerce: a three stage model. J. Retail Consum. Serv. 51, 320–330, 10.1016/j.jretconser.2019.07.004

[B57] Zhu W. Yan R. Ding Z. (2020). Analysing impulse purchasing in cross-border electronic commerce. Ind. Manage. Data Syst. 120, 1959–1974. 10.1108/IMDS-01-2020-0046

